# Supplementary material for: The causal role of circulating inflammatory markers in osteoporosis: a bidirectional Mendelian randomized study
Source: Front Immunol. 2024 Jul 18;15:1412298. doi: 10.3389/fimmu.2024.1412298 (PMC11291241; doi:10.3389/fimmu.2024.1412298)
Supplement: Supplementary file 1 [file DataSheet_1.zip › supplementary material/CIM-OSTEOPOROSIS /GCST90274831.csv_forest.pdf]

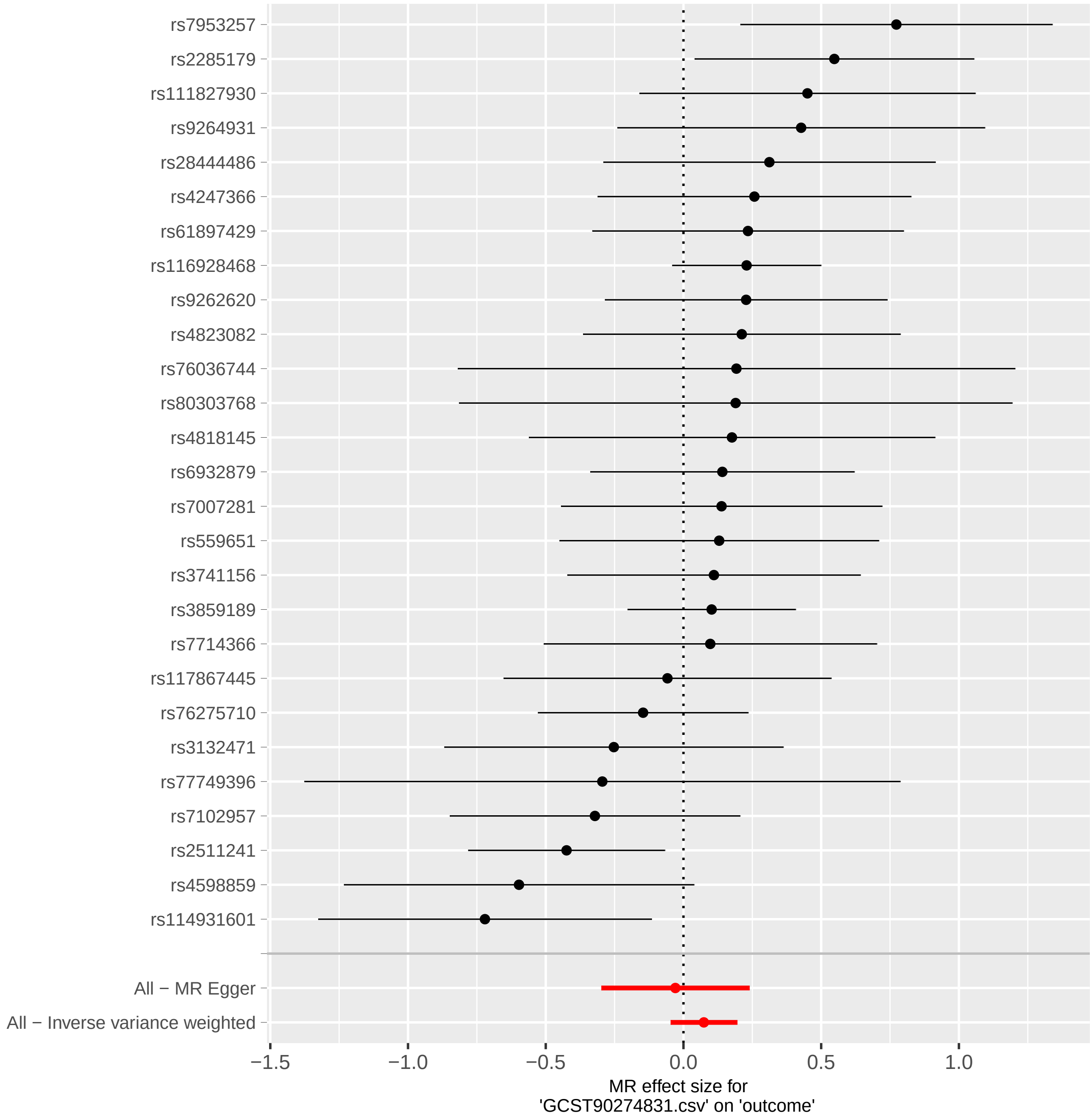
rs7953257
rs2285179
rs111827930
rs9264931
rs28444486
rs4247366
rs61897429
rs116928468
rs9262620
rs4823082
rs76036744
rs80303768
rs4818145
rs6932879
rs7007281
rs559651
rs3741156
rs3859189
rs7714366
rs117867445
rs76275710
rs3132471
rs77749396
rs7102957
rs2511241
rs4598859
rs114931601
All − MR Egger
All − Inverse variance weighted
−1.5
−1.0
−0.5
0.0
0.5
1.0
MR effect size for
'GCST90274831.csv' on 'outcome'
